# Cerebral baseline optical and hemodynamic properties in pediatric population: a large cohort time-domain near-infrared spectroscopy study

**DOI:** 10.1117/1.NPh.11.4.045009

**Published:** 2024-11-15

**Authors:** Valeria Calcaterra, Michele Lacerenza, Caterina Amendola, Mauro Buttafava, Davide Contini, Virginia Rossi, Lorenzo Spinelli, Sara Zanelli, Gianvincenzo Zuccotti, Alessandro Torricelli

**Affiliations:** a Buzzi Children’s Hospital, Pediatric Department, Milan, Italy; b University of Pavia, Pediatric and Adolescent Unit, Department of Internal Medicine, Pavia, Italy; c PIONIRS s.r.l., Milan, Italy; d Politecnico di Milano, Dipartimento di Fisica, Milan, Italy; e Consiglio Nazionale delle Ricerche, Istituto di Fotonica e Nanotecnologie, Milan, Italy; f University of Milan, Department of Biomedical and Clinical Science, Milan, Italy

**Keywords:** optical properties, hemodynamic properties, pediatric population, healthy, baseline, reproducibility

## Abstract

**Significance:**

Reference cerebral near-infrared spectroscopy (NIRS) data on the pediatric population are scarce, and in most cases, only cerebral oxygen saturation (${\mathrm{SO}}_{2}$) measured by continuous wave spatially resolved spectroscopy NIRS is reported. Absolute data for baseline optical and hemodynamic parameters are missing.

**Aim:**

We aimed at collecting baseline cerebral optical parameters [absorption coefficient, $\mu_{a}$; reduced scattering coefficient, $\mu_{s}^{\prime }$; differential pathlength factor (DPF)] and hemodynamic parameters [oxy-hemoglobin content (${\mathrm{HbO}}_{2}$), deoxyhemoglobin content (HHb), total hemoglobin content (tHB), ${\mathrm{SO}}_{2}$] in a large cohort of pediatric patients. The objectives are to establish reference optical values in this population and evaluate the reproducibility of a commercial time domain (TD) NIRS tissue oximeter.

**Approach:**

TD NIRS measurements were performed in the prefrontal cortex at 686 and 830 nm with a 2.5-cm source–detector distance and 1-Hz acquisition rate. Five independent measurements (after probe replacement) were taken for every subject. TD NIRS data were fitted to a photon diffusion model to estimate the optical parameters. From the absorption coefficients, the hemodynamic parameters were derived by Beer’s law. Auxological and physiological information was also collected to explore the potential correlations with NIRS data.

**Results:**

We measured 305 patients in the age range of 2 to 18 years. Absolute values for baseline optical and hemodynamic parameters were shown as a function of age and auxological variables. From the analysis of the repositioning after probe replacement, the time-domain near-infrared spectroscopy device exhibited an average precision (intended as coefficient of variation) of <5% for $\mu_{s}^{\prime }$, DPF, ${\mathrm{HbO}}_{2}$, HHb, and tHb, whereas precision was <2% for ${\mathrm{SO}}_{2}$.

**Conclusions:**

We provided baseline values for optical and hemodynamic parameters in a large cohort of healthy pediatric subjects with good precision, providing a foundation for future investigations into clinically relevant deviations in these parameters.

## 1 Introduction

The ability of red and near-infrared light (∼650 to 1000 nm) to diffuse into human tissues has fostered the development of a plethora of optical techniques to noninvasively study human brain functions and diseases.^1^ Near-infrared spectroscopy (NIRS) was initially introduced to monitor cerebral oxygen saturation (${\mathrm{SO}}_{2}$) in children and adults at the bedside by exploiting the different absorption spectra of oxygenated and deoxygenated hemoglobin.^2^^,^^3^ Later, functional NIRS took advantage of the neurovascular coupling mechanism, such as in functional magnetic resonance imaging, to provide a complementary tool to study human brain mapping in ecological settings.^4^^–^^6^ More recently, diffuse correlation spectroscopy (DCS), speckle contrast optical spectroscopy (SCOS), and interferometric NIRS completed the hemodynamic description by adding valuable information on cerebral perfusion and metabolism.^7^

Neonates, infants, and children, having thinner skulls than adults, show reduced light attenuation and enhanced light penetration; therefore, they represent the ideal target population for probing the brain through noninvasive optical techniques. Nonetheless, several studies are currently performed also on adults. The incessant growth of these optical techniques and their adoption in biomedical and clinical applications has been supported by the availability of guidelines for best practice and (open source) data analysis tools^8^^,^^9^ and by the parallel advance in modeling light propagation in diffusive media.^10^^,^^11^

Although for several applications the measurement of trends, or relative changes with respect to a baseline (in arbitrary units), might be sufficient (e.g., when studying the hemodynamic response function following a stimulus or the perfusion changes during a bed tilt test), the knowledge of the absolute baseline values for the quantities of interest is crucial when it comes to quantifying those changes in view of a more accurate and robust assessment of the response.

Aiming for quantitation, the knowledge of tissue optical properties (absorption coefficient, $\mu_{a}$, and reduced scattering coefficient, $\mu_{s}^{\prime }$) is fundamental because it enables accurate modeling of light propagation in complex heterogeneous structures (such as the human head) and the investigation of specific features of the optical techniques (e.g., depth penetration, depth sensitivity, signal-to-noise ratio, and contrast-to-noise ratio).

In the literature, several studies report optical properties of neonates^12^^–^^20^ and adults,^21^^–^^32^ whereas very few focus on the pediatric population with also a limited number of measured subjects.^33^^–^^36^ NIRS data on population in pediatric age in the majority of cases refer only to ${\mathrm{SO}}_{2}$, as measured by continuous wave (CW) spatially resolved spectroscopy NIRS devices, whereas absolute data for baseline optical and hemodynamic parameters are missing.^37^

The aim of this work is to collect baseline cerebral optical parameters [absorption coefficient, $\mu_{a}$; reduced scattering coefficient, $\mu_{s}^{\prime }$; and differential pathlength factor (DPF)] and hemodynamic parameters [oxy-hemoglobin content (${\mathrm{HbO}}_{2}$), deoxyhemoglobin content (HHb), total hemoglobin content ($\mathrm{tHb}=\mathrm{HHb}+{\mathrm{HbO}}_{2}$; ${\mathrm{SO}}_{2}={\mathrm{HbO}}_{2}/\mathrm{tHb}$)] in a large cohort of pediatric patients in the age range of 2 to 18 years. The primary objective is to establish reference optical and hemodynamic values in this population, whereas a secondary (but equally important) objective is to evaluate the reproducibility of a time domain (TD) NIRS tissue oximeter.

## 2 Material and Methods

### 2.1 Subjects

The study was conducted from March 2023 to February 2024 at Buzzi Children’s Hospital (Milan, Italy) on a pediatric cohort of healthy subjects in stable conditions. The following inclusion criteria were considered: absence of fever, absence of cardiac or pulmonary pathologies, no chronic diseases, no ongoing pharmacological treatments, stable vital parameters [heart rate (HR), respiratory frequency (BR), and peripheral oxygen saturation (${\mathrm{SpO}}_{2}$)], absence of wound in the measured position, and confirmation of normal hematocrit levels through blood analyses. The study was conducted in accordance with the Helsinki Declaration of 1975, as revised in 2008. The institutional ethics committee approved the protocol (Ethics Committee Milano Area 1; Study Registration 2022/ST/229; Protocol No. 0004021/2023 Date 30/01/2023). After receiving information about the study, all participants, or their guardians, provided written consent.

### 2.2 Time-Domain Near-Infrared Spectroscopy (TD-NIRS) Device

**Fig. 1:**
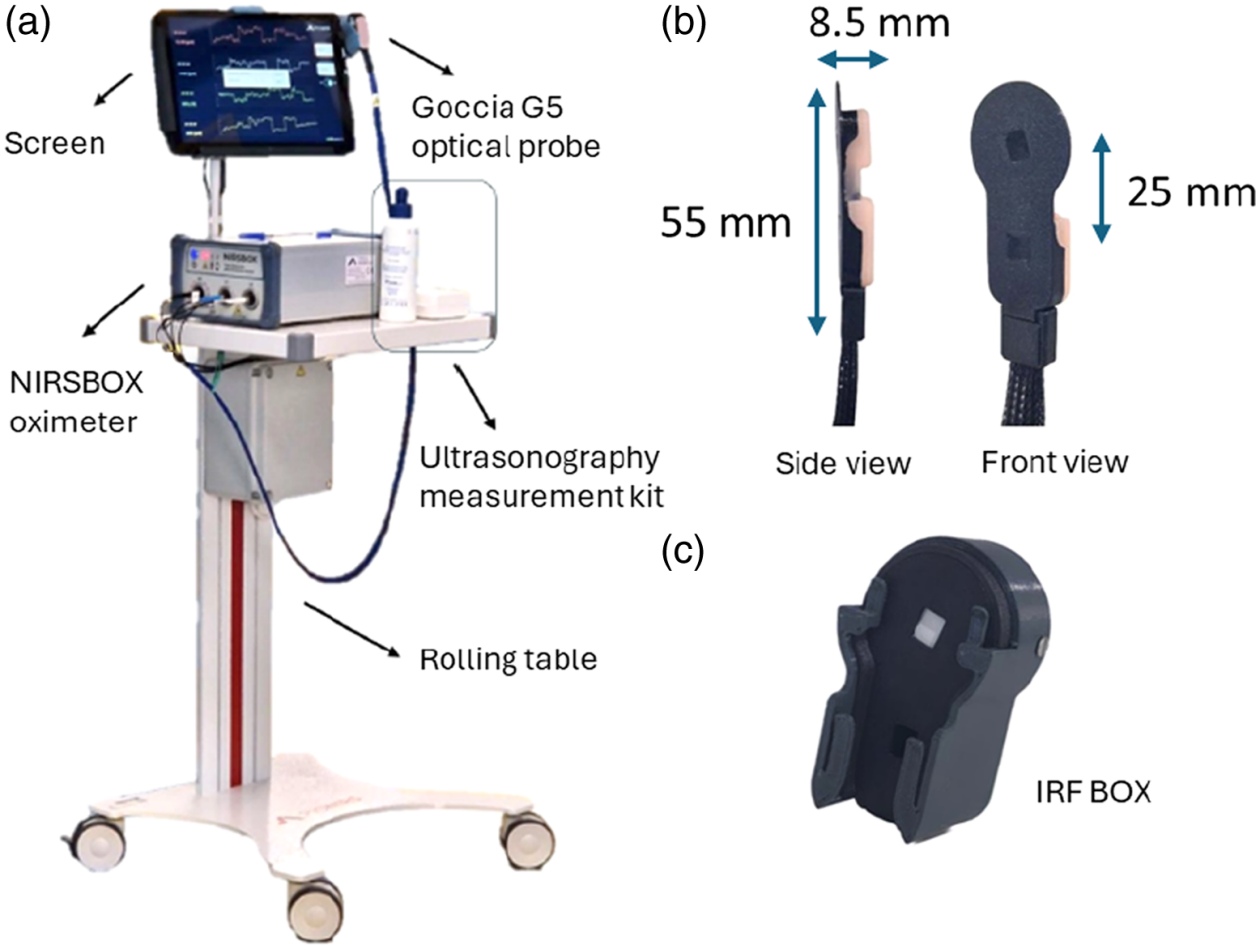
(a) NIRSBOX tissue oximeter, as used in the clinical environment (cart-mounted configuration). (b) G5 Goccia probe. (c) IRF box.

A commercially available, research-grade, tissue oximeter, NIRSBOX (PIONIRS s.r.l., Milan, Italy), based on TD-NIRS technology was used (see Fig. 1).^38^ The device employs proprietary picosecond diode lasers emitting at 686 and 830 nm, along with a single-photon detector (silicon photomultiplier, with optical filters to reduce ambient light noise) and timing electronics (time-to-digital converter, with a 9.7-ps/ch resolution) to record the distribution of time-of-flight (DTOF) for the photons re-emitted from the tissue. The NIRSBOX device is battery-operated (7-h lifetime) and hosted in a compact, four-wheel medical grade chart equipped with a 13-in. screen. In this study, the G5 Goccia optical probe (PIONIRS s.r.l., Milan, Italy) was employed, characterized by a single channel with a source–detector distance ρ=2.5  cm and a built-in capacitive contact sensor to ensure accurate application on the tissue and secure enablement of laser emission. The probe is flexible, waterproof, and undergoes sanitation with isopropyl alcohol among each patient. To acquire the instrument response function (IRF), the probe is positioned into the PIONIRS IRF box.^39^

### 2.3 Protocol

Measurements were performed on the left prefrontal cortex (Fp1 position of the 10/20 International System Mapping), targeting cerebral optical properties and hemodynamic parameters. The protocol included the acquisition of five DTOFs at a 1-Hz acquisition frequency. Then, other four identical measurements were performed after probe replacement (i.e., removing the probe from the tissue, placing the probe again in contact with the tissue, and acquiring data). Overall, 25 DTOFs were acquired (five replacements times five DTOFs/replacement) from each subject. The optical probe was manually held in place by the clinical operator during measurement and kept in hand during probe removal and probe replacement. The entire measurement protocol took ∼2  min per subject. To assess a child’s growth and physical development, in all children, auxological measurements including weight, height, body mass index (BMI), and head circumference were recorded. BMI values were calculated as body weight (kg) divided by squared height (m^2^) and then standardized into BMI z-scores according to the reference values obtained from the World Health Organization database,^40^ specifically “BMI-for-age 2 to 5 years” and “BMI-for-age 5 to 19 years.”^41^^,^^42^ In addition, following the usual clinical practice, HR, BR, and ${\mathrm{SpO}}_{2}$ were measured by standard clinical grade devices, whereas hematocrit percentage (HTC) and hemoglobin concentration (Hb) were obtained by venous sample.

### 2.4 Data Analysis

The solution of the photon diffusion equation for a semi-infinite homogeneous medium with extrapolated boundary conditions was used^43^ (after convolution with the IRF) to retrieve from each measured DTOF the optical properties (absorption coefficient, $\mu_{a}$, and reduced scattering coefficient, $\mu_{s}^{\prime }$) of the tissue under investigation. Then, from each DTOF, the DPF was calculated as DPF(λ)=v⟨t(λ)⟩/ρ, where ⟨t(λ)⟩ is the photon mean time of flight, v=c/n is the speed of light in vacuum, n=1.4 is the tissue refractive index (assumed constant), and λ is the wavelength.

From $\mu_{a}$ at 686 and 830 nm by exploiting the Beer law,^44^ ${\mathrm{HbO}}_{2}$ and HHb were obtained assuming hemoglobin as the unique chromophore contributing to absorption. Hence, tHb and ${\mathrm{SO}}_{2}$ were calculated. Unless differently specified, for each subject, the optical and hemodynamic parameters were averaged over all the 25 acquired DTOFs.

### 2.5 Statistical Analysis

The correlation (linear relationship) between two variables has been evaluated by computing the Pearson correlation coefficient r and the corresponding p-value. We considered no correlation for |r|<0.3, low correlation 0.3≤|r|<0.5, moderate correlation for 0.5≤|r|<0.7, and high correlation for 0.7≤|r|<1.

## 3 Results

### 3.1 Demographic and Clinical Features of the Measured Subjects

A total of 307 healthy participants aged between 2 and 18 years were enrolled over 11 months. Two subjects initially enrolled were later excluded (not compliant due to intense crying during the measurements), resulting in 305 subjects. As shown in Table 1, the population is uniformly distributed according to gender and age, with 52% females (9.6±4.6 years) and 48% males (9.5±4.2 years).

**Table 1:** Enrolled subjects per age and gender.

Age (years)	Female (no.)	Male (no.)	Total (no.)	Female (%)	Male (%)	Total (%)
2 to 4	20	23	43	13	16	14
4 to 6	21	16	37	13	11	12
6 to 8	25	18	43	16	12	14
8 to 10	17	19	36	11	13	12
10 to 12	17	21	38	11	14	12
12 to 14	22	27	49	14	18	16
14 to 16	22	14	36	14	9	12
16 to 18	13	10	23	8	7	8
Total	157	148	305	100	100	100

Demographic and auxological descriptors are reported in Table 2. Data do not show abnormal trends. As expected, there is a strong correlation between age and head circumference (Pearson’s correlation coefficient of 0.7 both for females and males) and a moderate correlation between age and BMI (Pearson’s correlation coefficient of 0.5 and 0.6 for females and males, respectively).

**Table 2:** Auxological descriptors (average ± standard deviation) of the subjects per age and gender.

Age (years)	BMI ($\mathrm{kg}/m^{2}$)		BMI z-score		Head circumference (cm)	
	Female	Male	Female	Male	Female	Male
2 to 4	15.0 ± 1.5	15.4 ± 1.5	−0.3 ± 1.0	−0.1 ± 1.2	48.9 ± 1.6	50.4 ± 1.5
4 to 6	14.8 ± 1.7	15.2 ± 1.8	−0.3 ± 1.0	−0.1 ± 1.3	50.8 ± 1.6	52.1 ± 1.3
6 to 8	16.7 ± 3.5	17.4 ± 6.0	0.7 ± 1.9	1.1 ± 3.6	52.2 ± 2.1	52.2 ± 1.8
8 to 10	19.6 ± 5.3	17.8 ± 3.5	1.5 ± 2.3	0.9 ± 1.9	52.8 ± 2.3	53.1 ± 1.4
10 to 12	19.6 ± 5.4	18.7 ± 3.3	0.9 ± 2.0	0.8 ± 1.5	53.5 ± 2.0	54.2 ± 1.8
12 to 14	22.6 ± 7.4	23.9 ± 7.1	1.4 ± 2.5	2.3 ± 2.8	54.7 ± 2.0	55.3 ± 1.8
14 to 16	22.4 ± 6.7	23.3 ± 4.8	0.6 ± 2.0	1.2 ± 1.5	54.9 ± 2.2	55.7 ± 2.9
16 to 18	23.1 ± 4.8	24.0 ± 6.4	0.6 ± 1.4	0.9 ± 2.0	55.9 ± 1.4	56.9 ± 2.8
Total	19.0 ± 5.9	19.3 ± 5.8	0.6 ± 1.9	0.9 ± 2.3	52.8 ± 2.9	53.5 ± 2.7

### 3.2 Physiological Descriptors of the Measured Subjects

Table 3 shows the physiological descriptors (average ± standard deviation) of the subjects per age cluster and gender. Overall, no abnormal values for all parameters were recorded. The average HTC ranges from 34.3% to 41.9%. A moderate decrease of HR and BR with age is observed, as expected (Pearson’s correlation coefficient for HR: r=−0.6 with $p\text{-}\text{value}=1\times 10^{-15}$ and r=−0.5 with $p\text{-}\text{value}=2\times 10^{-09}$, for females and males, respectively; Pearson’s correlation coefficient for BR: r=−0.4 both for females and males with $p\text{-}\text{value}=5\times 10^{-08}$ and $1\times 10^{-07}$, respectively). The average ${\mathrm{SpO}}_{2}$ is higher than 98.1% with no correlation with age. As expected, HTC and Hb are strongly correlated (Pearson’s correlation coefficient of 0.9 both for females and males with $p\text{-}\text{value}=3\times 10^{-71}$ and $4\times 10^{-60}$, respectively), whereas a low correlation of both HTC and Hb with age is observed (Pearson’s correlation coefficient for HTC: r=0.3 with $p\text{-}\text{value}=1\times 10^{-04}$ and r=0.4 with $p\text{-}\text{value}=1\times 10^{-08}$ for females and males, respectively; Pearson’s correlation coefficient for Hb: r=0.3 with $p\text{-}\text{value}=1\times 10^{-04}$ and r=0.4 with $p\text{-}\text{value}=5\times 10^{-07}$ for females and males, respectively).

**Table 3:** Physiological descriptors (average ± standard deviation) of the subjects per age and gender. HTC, hematocrit content (%); Hb, hemoglobin content (g/dl); HR, heart rate (beat/min); BR, breathing rate (breath/min); ${\mathrm{SpO}}_{2}$, arterial oxygen saturation (%).

Age (years)	HTC (%)		Hb (g/dl)		HR (beat/min)		BR (breath/min)		${\mathrm{SpO}}_{2}$ (%)	
	Female	Male	Female	Male	Female	Male	Female	Male	Female	Male
2 to 4	34.3 ± 2.7	35.4 ± 3.5	11.6 ± 0.8	12.1 ± 1.2	112.0 ± 12.3	104.7 ± 15.7	25.8 ± 3.6	24.8 ± 3.2	98.8 ± 1.3	98.5 ± 1.3
4 to 6	34.3 ± 2.0	36.5 ± 3.7	11.8 ± 0.8	12.9 ± 1.3	103.4 ± 10.3	98.8 ± 14.0	23.0 ± 2.3	23.7 ± 3.4	98.7 ± 1.2	98.6 ± 0.8
6 to 8	36.4 ± 3.9	37.8 ± 4.3	12.3 ± 1.4	12.9 ± 1.7	88.4 ± 12.3	95.2 ± 16.9	23.0 ± 3.2	23.3 ± 4.2	98.5 ± 1.3	98.1 ± 1.0
8 to 10	36.1 ± 3.3	36.4 ± 2.3	12.5 ± 1.1	12.8 ± 1.0	93.6 ± 17.7	87.9 ± 10.6	24.2 ± 5.0	21.5 ± 2.7	98.8 ± 1.0	98.3 ± 1.3
10 to 12	38.5 ± 2.8	37.5 ± 3.4	13.3 ± 1.0	13.0 ± 1.3	85.9 ± 13.8	88.4 ± 8.7	21.8 ± 3.5	22.2 ± 2.9	98.4 ± 0.9	98.2 ± 0.9
12 to 14	38.5 ± 3.6	39.3 ± 3.1	13.2 ± 1.3	13.4 ± 1.2	83.4 ± 7.4	87.4 ± 15.0	20.0 ± 2.0	21.7 ± 3.7	99.0 ± 1.1	98.6 ± 1.0
14 to 16	37.3 ± 4.1	39.8 ± 2.6	12.6 ± 1.5	13.9 ± 0.7	78.6 ± 12.3	84.1 ± 15.6	20.2 ± 2.1	19.9 ± 2.3	99.2 ± 0.8	98.4 ± 1.3
16 to 18	36.4 ± 3.7	41.9 ± 3.2	12.5 ± 1.4	14.1 ± 0.9	83.5 ± 13.6	78.1 ± 9.5	20.8 ± 6.0	19.8 ± 2.4	98.5 ± 1.2	97.9 ± 0.7
Total	36.5 ± 3.7	37.8 ± 3.8	12.5 ± 1.3	13.0 ± 1.3	91.2 ± 16.5	91.6 ± 15.8	22.4 ± 4.0	22.3 ± 3.6	98.7 ± 1.1	98.4 ± 1.1

### 3.3 Optical Properties of the Measured Subjects

Table 4 reports the optical properties (average ± standard deviation) of the subjects at 686 and 830 nm per age (2-year clusters) and gender. For all variables, no differences can be found between the female and male groups at any age. The average $\mu_{a}$ values range from 0.16 to $0.25\;{\mathrm{cm}}^{-1}$ at 686 nm and from 0.17 to $0.24\;{\mathrm{cm}}^{-1}$ at 830 nm with an overall dispersion (as measured by the coefficient of variation over the entire population) of 21% at 686 nm and 18% at 830 nm. As expected, according to the empirical approximation of Mie’s theory,^45^ $\mu_{s}^{\prime }$ is higher at 686 than at 830 nm with average values ranging from 13.2 to $14.4\;{\mathrm{cm}}^{-1}$ at 686 nm and from 11.3 to $12.3\;{\mathrm{cm}}^{-1}$ at 830 nm. Interestingly, the dispersion for scattering data is lower (almost half) than the dispersion for the absorption data with 10% at 686 nm and 9% at 830 nm. The average DPF varies from 4.2 to 5.2 at 686 nm and from 3.8 to 4.8 at 830 nm with 16% and 12% dispersion at 686 and 830 nm, respectively.

**Table 4:** Optical properties (average ± standard deviation) of the subjects at 686 and 830 nm per age and gender. Absorption coefficient, $\mu_{a}$ (${\mathrm{cm}}^{-1}$), reduced scattering coefficient $\mu_{s}^{\prime }$ (${\mathrm{cm}}^{-1}$) DPF (−).

Age (years)	$\mu_{a}$ (686 nm)		$\mu_{a}$ (830 nm)		$\mu_{s}^{\prime }$ (686 nm)		$\mu_{s}^{\prime }$ (830 nm)		DPF (686 nm)		DPF (830 nm)	
	Female	Male	Female	Male	Female	Male	Female	Male	Female	Male	Female	Male
2 to 4	0.22 ± 0.03	0.24 ± 0.03	0.21 ± 0.02	0.23 ± 0.02	13.2 ± 1.4	14.0 ± 1.4	11.3 ± 1.2	11.9 ± 1.0	4.3 ± 1.3	4.2 ± 0.4	4.0 ± 0.4	3.9 ± 0.3
4 to 6	0.24 ± 0.03	0.25 ± 0.03	0.22 ± 0.03	0.24 ± 0.03	13.7 ± 1.0	14.0 ± 1.0	11.7 ± 0.7	12.1 ± 0.8	4.2 ± 0.4	4.2 ± 0.4	4.0 ± 0.3	3.9 ± 0.4
6 to 8	0.23 ± 0.04	0.24 ± 0.04	0.21 ± 0.03	0.23 ± 0.04	13.5 ± 1.3	13.7 ± 1.1	11.6 ± 1.0	11.9 ± 0.9	4.3 ± 0.3	4.2 ± 0.5	4.0 ± 0.4	4.0 ± 0.5
8 to 10	0.23 ± 0.03	0.22 ± 0.03	0.22 ± 0.03	0.21 ± 0.02	13.7 ± 0.9	13.5 ± 1.3	11.8 ± 0.8	11.7 ± 1.0	4.3 ± 0.4	4.3 ± 0.5	3.9 ± 0.3	4.0 ± 0.4
10 to 12	0.22 ± 0.06	0.22 ± 0.04	0.22 ± 0.05	0.22 ± 0.03	13.6 ± 1.1	14.0 ± 1.0	11.9 ± 0.8	12.0 ± 0.9	4.4 ± 0.6	4.6 ± 0.8	4.1 ± 0.7	4.0 ± 0.4
12 to 14	0.20 ± 0.04	0.23 ± 0.05	0.21 ± 0.04	0.24 ± 0.05	13.7 ± 1.2	14.2 ± 1.4	11.9 ± 0.9	12.3 ± 1.2	4.7 ± 0.6	4.5 ± 0.6	4.2 ± 0.4	4.0 ± 0.5
14 to 16	0.18 ± 0.03	0.23 ± 0.06	0.18 ± 0.03	0.25 ± 0.07	14.0 ± 1.4	14.4 ± 1.6	12.0 ± 1.2	12.2 ± 1.3	5.1 ± 0.7	4.6 ± 0.8	4.6 ± 0.6	3.8 ± 0.6
16 to 18	0.16 ± 0.04	0.20 ± 0.06	0.17 ± 0.03	0.21 ± 0.05	13.5 ± 1.0	13.9 ± 2.5	11.9 ± 0.8	11.8 ± 1.6	5.2 ± 0.6	4.9 ± 1.1	4.8 ± 0.4	4.1 ± 0.7
Total	0.21 ± 0.05	0.23 ± 0.05	0.21 ± 0.04	0.23 ± 0.04	13.6 ± 1.2	14.0 ± 1.4	11.7 ± 1.0	12.0 ± 1.1	4.6 ± 0.8	4.4 ± 0.7	4.2 ± 0.5	4.0 ± 0.5

**Fig. 2:**
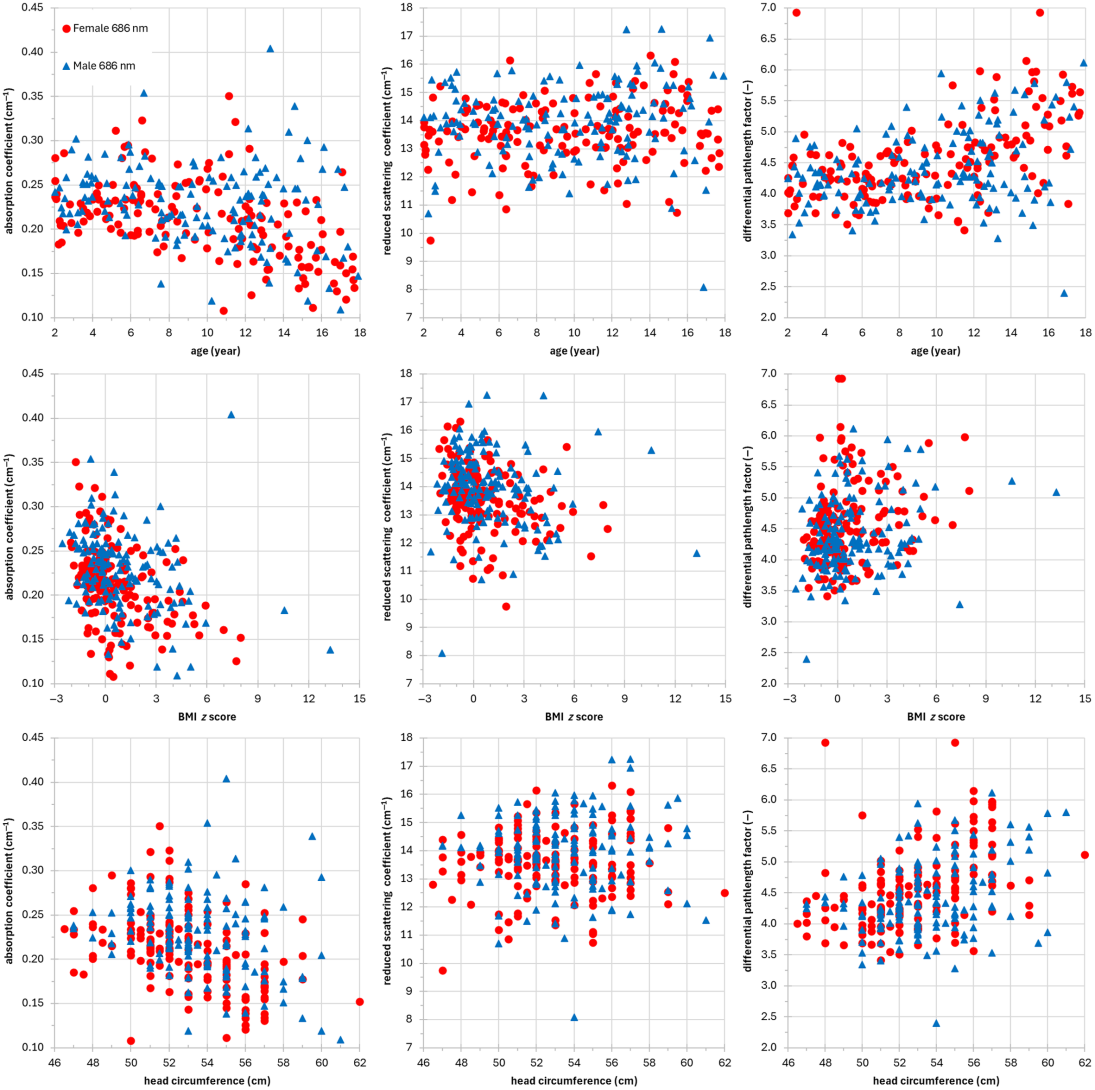
Optical properties of the subjects (female, red circles; male, blue triangles) at 686 nm as a function of age (left column), BMI z-score (middle column), and head circumference (right column). Top row: absorption coefficient (${\mathrm{cm}}^{-1}$), middle row: reduced scattering coefficient (${\mathrm{cm}}^{-1}$), and bottom row: differential pathlength factor.

**Fig. 3:**
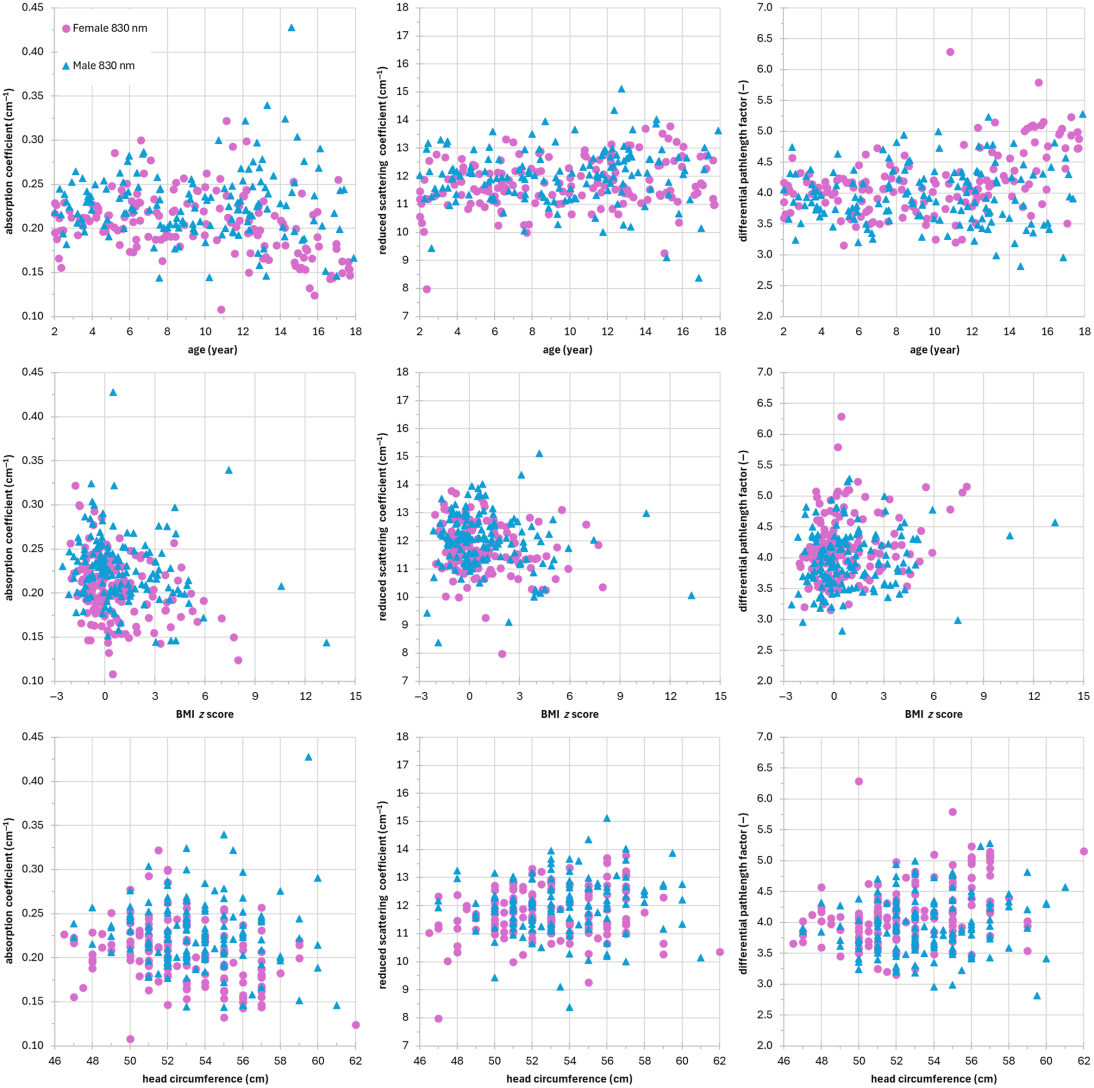
Optical properties of the subjects (female, purple squares; male, cyan diamonds) at 830 nm as a function of age (left column), BMI z-score (middle column), and head circumference (right column). Top row: absorption coefficient (${\mathrm{cm}}^{-1}$), middle row: reduced scattering coefficient (${\mathrm{cm}}^{-1}$), and bottom row: differential pathlength factor.

Figures 2 and 3 show the optical properties of the subjects (females and males at 686 and 830 nm, respectively) as a function of age, BMI z-score, and head circumference. Each dot represents a subject. As a general comment, we can notice the presence of few outliers in the distributions of the optical parameters. To evaluate the presence of correlations, we have reported in Table 5 the Pearson’s correlation coefficient r and the related p-value. We have a moderate/low inverse correlation with age for $\mu_{a}$ in females (r=−0.5 and −0.3 with $p\text{-}\text{value}=4\times 10^{-11}$ and $2\times 10^{-05}$ at 686 and 830 nm, respectively) and no correlation in males. No correlation with age is found for $\mu_{s}^{\prime }$ for both females and males. There is a moderate/low correlation (r=0.5 and 0.4 with $p\text{-}\text{value}=8\times 10^{-12}$ and $4\times 10^{-09}$ at 686 and 830 nm, respectively) with age for DPF in females, whereas there is a low correlation only at 686 nm (r=0.3 with $p\text{-}\text{value}=9\times 10^{-05}$) for DPF in males. Regarding the correlation with BMI z-score, we have a low inverse correlation for $\mu_{a}$ in females (r=−0.4 and −0.3 with $p\text{-}\text{value}=1\times 10^{-07}$ and $2\times 10^{-05}$ at 686 and 830 nm, respectively) and a low correlation in males only at 686 nm (r=−0.3 with $p\text{-}\text{value}=2\times 10^{-04}$). No correlation with BMI z-score for $\mu_{s}^{\prime }$ is observed in males, whereas a low correlation at 686 nm is found in females (r=−0.3 with $p\text{-}\text{value}=4\times 10^{-04}$). Finally, no correlation with BMI z-score for DPF is found in the female and male groups. A moderate or low inverse correlation (r=−0.5 and −0.3 with $p\text{-}\text{value}=3\times 10^{-09}$ and $6\times 10^{-05}$ at 686 nm and 830 nm, respectively) with head circumference is observed for $\mu_{a}$ in females, whereas we have a low inverse correlation only at 686 nm (r=−0.3 with $p\text{-}\text{value}=6\times 10^{-04}$) in males. Again, no correlation is found for $\mu_{s}^{\prime }$ in both the female and male groups. A low correlation with head circumference for DPF in females (r=0.4 both at 686 and 830 nm with $p-\mathrm{value}=1\times 10^{-8}$ and $1\times 10^{-06}$, respectively) and a low correlation in males at 686 nm (r=0.3 with $p\text{-}\text{value}=2\times 10^{-5}$) are observed.

**Table 5:** Pearson’s correlation coefficient r and corresponding p-value for optical properties (bold 0.5≤|r|<0.7 moderate correlation, italics 0.3≤|r|<0.5 low correlation, no emphasis 0≤|r|<0.3 no correlation, and bold italics p-value≥0.05).

		$\mu_{a}$ (686 nm)		$\mu_{a}\;$ (830 nm)		$\mu_{s}^{\prime }\;$ (686 nm)		$\mu_{s}^{\prime }$ (830 nm)		DPF (686 nm)		DPF (830 nm)	
		Female	Male	Female	Male	Female	Male	Female	Male	Female	Male	Female	Male
Age	r	**−0.5**	−0.2	*−0.3*	0.0	0.1	0.1	0.2	0.1	**0.5**	*0.3*	*0.4*	0.0
	p-value	$4\times 10^{-11}$	$5\times 10^{-03}$	$2\times 10^{-05}$	$7\times 10^{-01}$	$2\times 10^{-01}$	$4\times 10^{-01}$	$5\times 10^{-03}$	$5\times 10^{-01}$	$8\times 10^{-12}$	$9\times 10^{-05}$	$4\times 10^{-09}$	$7\times 10^{-01}$
BMI z-score	r	*−0.4*	*−0.3*	*−0.3*	−0.2	*−0.3*	−0.2	−0.2	−0.1	0.2	0.2	0.2	0.1
	p-value	$1\times 10^{-07}$	$2\times 10^{-04}$	$2\times 10^{-05}$	$2\times 10^{-02}$	$4\times 10^{-04}$	$4\times 10^{-02}$	$1\times 10^{-02}$	$2\times 10^{-01}$	$2\times 10^{-03}$	$5\times 10^{-03}$	$7\times 10^{-03}$	$9\times 10^{-02}$
Head circ.	r	**−0.5**	*−0.3*	*−0.3*	0.0	0.1	0.0	0.2	0.1	*0.4*	*0.3*	*0.4*	0.1
	p-value	$3\times 10^{-09}$	$6\times 10^{-04}$	$6\times 10^{-05}$	$8\times 10^{-01}$	$4\times 10^{-01}$	$6\times 10^{-01}$	$6\times 10^{-02}$	$2\times 10^{-01}$	$1\times 10^{-08}$	$2\times 10^{-05}$	$1\times 10^{-06}$	$1\times 10^{-01}$

**Fig. 4:**
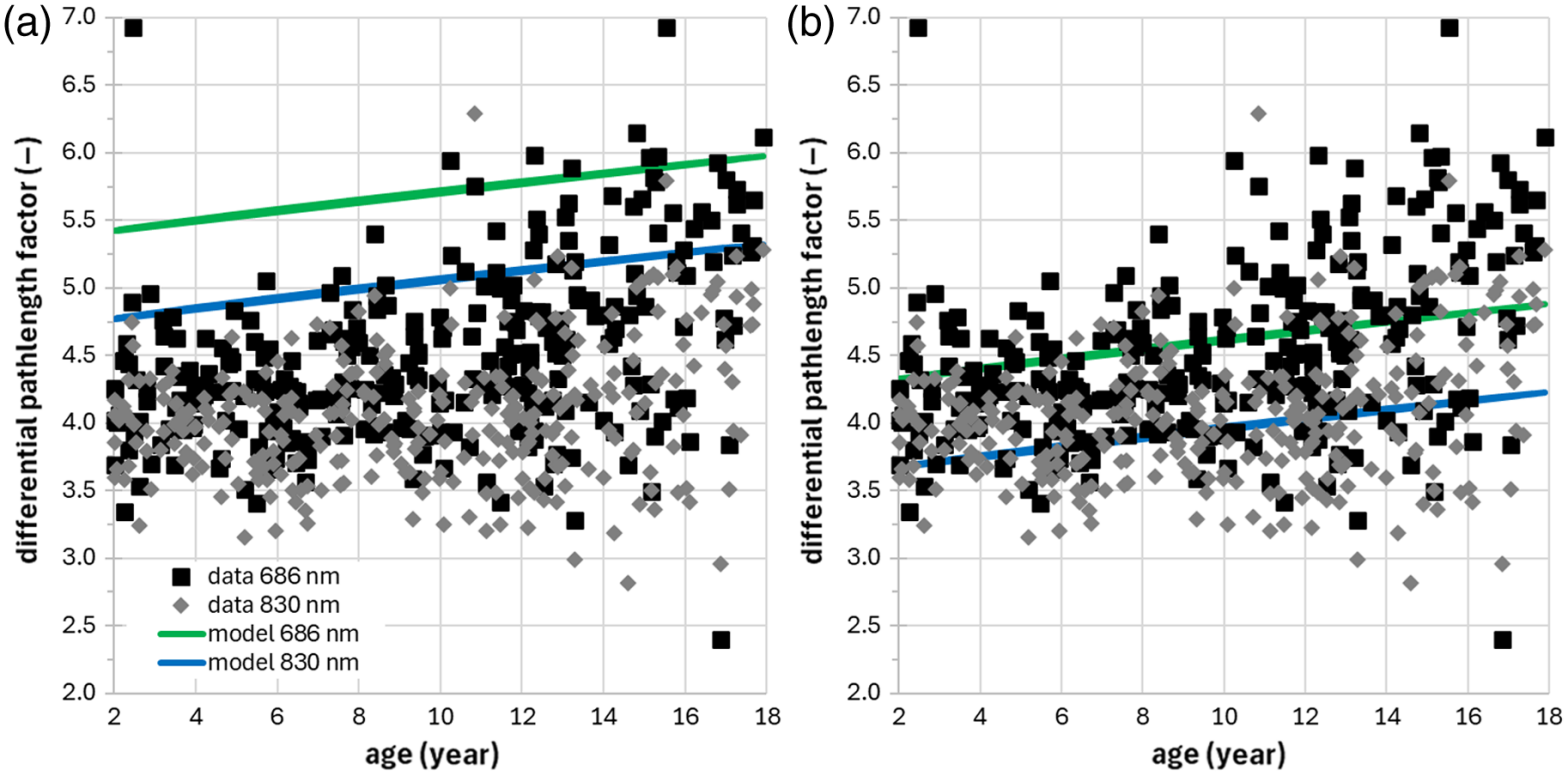
DPF at 686 and 830 nm as a function of age and estimates obtained by the general equation^47^ with α=223.3 (a) and with α=222.2 (b).

We have compared the results for the DPF (with no distinction between females and males) with the general equation $\mathrm{DPF}(A,\lambda )=\alpha +\beta A^{\gamma }+\delta \lambda^{3}+\varepsilon \lambda^{3}+\zeta \lambda$, where A is the age in years and λ the wavelength in nanometers, derived by Scholkmann and Wolf.^46^ When using the original values for parameters α−ζ, the agreement is not perfect as shown in Fig. 4(a). However, we notice that with only a slight change (<0.5%) from α=223.3 to α=222.2 (obtained by minimizing the error between the DPF model and data), the agreement improves as shown in Fig. 4(b). However, the correlation is low or null because the Pearson correlation coefficient is 0.3 and 0.2, respectively at 686 nm at 830 nm, probably affected by some outliers.

### 3.4 Hemodynamic Properties of the Measured Subjects

Table 6 shows the hemodynamic parameters (average ± standard deviation) of the subjects per age cluster and gender. For all variables, no differences can be found between the female and male groups at any age. The average ${\mathrm{HbO}}_{2}$ and HHb values range from 55.9 to 86.0  μM and from 25.4 to 39.3  μM, respectively, with an overall dispersion of 18% and 23%. These values result in an average tHb ranging from 81.3 to 120.0  μM with 18% dispersion and average ${\mathrm{SO}}_{2}$ in the range from 64.3% to 71.8% with 6% dispersion.

**Table 6:** Hemodynamic properties (average ± standard deviation) of the subjects per age and gender.

Age (years)	${\mathrm{HbO}}_{2}$ (μM)		HHb (μM)		tHb (μM)		${\mathrm{SO}}_{2}$ (%)	
	Female	Male	Female	Male	Female	Male	Female	Male
2 to 4	65.9 ± 8.2	72.6 ± 8.0	36.3 ± 5.5	39.3 ± 5.0	102.2 ± 10.9	111.8 ± 10.9	64.5 ± 3.9	64.9 ± 3.1
4 to 6	69.4 ± 8.7	77.3 ± 9.7	38.5 ± 6.0	39.2 ± 5.4	107.9 ± 13.3	116.6 ± 13.5	64.3 ± 2.8	66.3 ± 2.8
6 to 8	69.5 ± 11.8	74.4 ± 12.2	36.4 ± 6.3	37.5 ± 7.9	105.9 ± 16.8	112.0 ± 17.9	65.5 ± 3.1	66.6 ± 3.6
8 to 10	72.3 ± 8.5	70.4 ± 8.7	36.5 ± 5.7	35.1 ± 5.5	108.8 ± 12.9	105.4 ± 11.0	66.5 ± 3.0	66.7 ± 4.3
10 to 12	74.0 ± 14.3	73.9 ± 12.1	34.7 ± 10.4	33.8 ± 7.0	108.8 ± 23.9	107.7 ± 16.3	68.5 ± 3.4	68.7 ± 4.5
12 to 14	70.1 ± 12.0	80.1 ± 15.4	30.2 ± 6.2	35.7 ± 9.3	100.3 ± 17.2	115.8 ± 22.7	70.0 ± 3.1	69.3 ± 3.9
14 to 16	61.7 ± 11.6	86.0 ± 24.1	27.5 ± 5.8	34.0 ± 10.8	89.2 ± 16.0	120.0 ± 31.7	69.2 ± 3.9	71.8 ± 6.0
16 to 18	55.9 ± 9.4	73.5 ± 15.6	25.4 ± 6.5	30.2 ± 11.2	81.3 ± 15.0	103.7 ± 24.1	69.0 ± 3.6	71.5 ± 5.9
Total	67.7 ± 11.9	75.9 ± 14.2	33.5 ± 7.9	36.0 ± 8.2	101.2 ± 18.3	111.9 ± 19.5	67.1 ± 4.0	67.9 ± 4.8

**Fig. 5:**
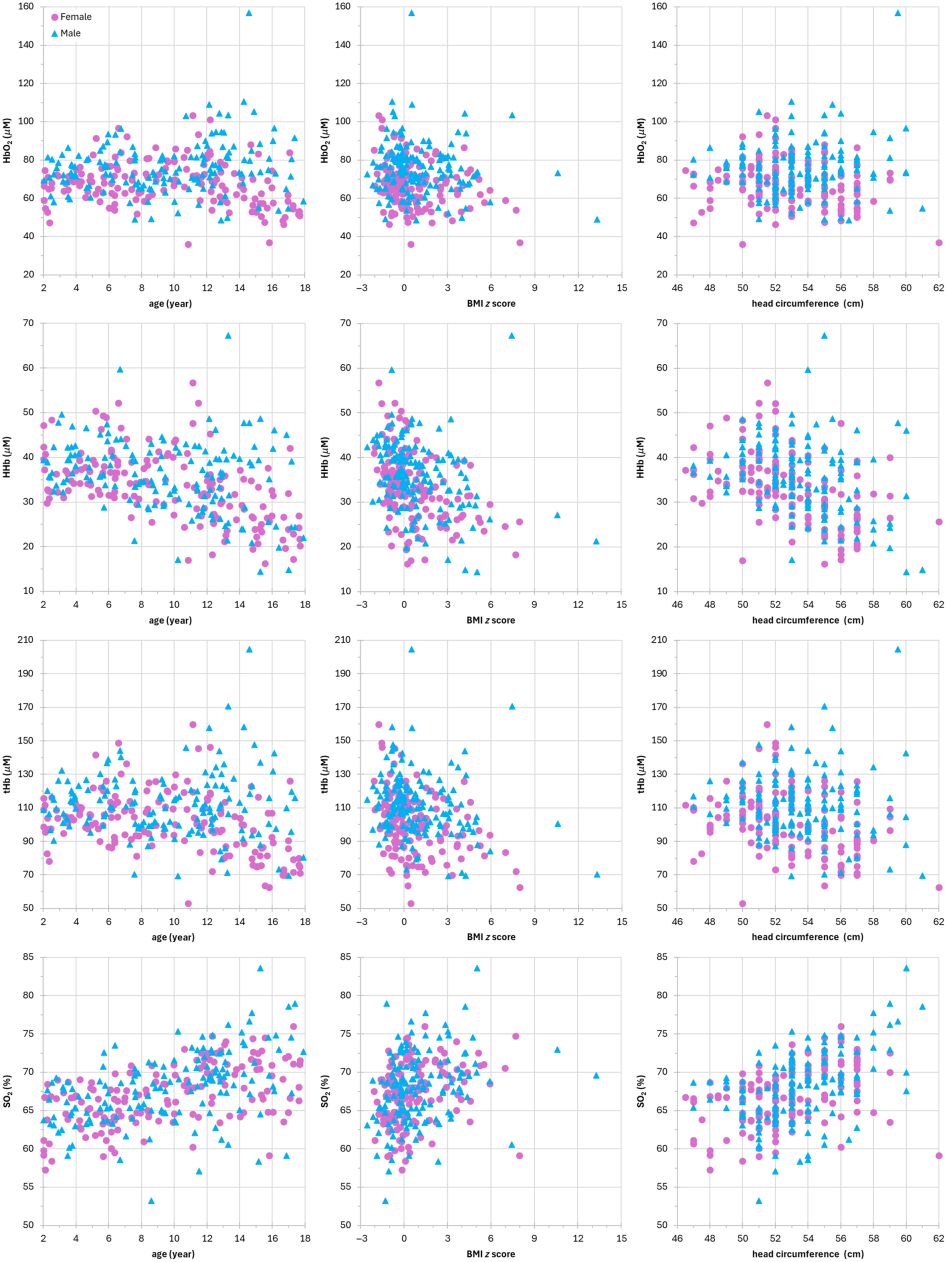
Hemodynamic parameters of the subjects (female, purple circle; male, cyan triangle) as a function of age (left column), BMI z-score (middle column), and head circumference (right column). Rows from top to bottom: ${\mathrm{HbO}}_{2}$ (μM), HHb (μM), tHb (μM), and ${\mathrm{SO}}_{2}$ (%).

Figure 5 shows the hemodynamic parameters of the subjects (female and male) as a function of age, BMI z-score, or head circumference, whereas in Table 7, we report the corresponding Pearson’s correlation coefficients r and p-values. No correlation with age for ${\mathrm{HbO}}_{2}$ is found for both females and males. Conversely, moderate (r=−0.5 with $p\text{-}\text{value}=1\times 10^{-12}$) inverse correlation with age is found for HHb in females and low inverse correlation (r=−0.3 with $p\text{-}\text{value}=3\times 10^{-04}$) in males. Low (r=−0.4 with $p\text{-}\text{value}=3\times 10^{-06}$) inverse correlation with age is present for tHb in females only, whereas a moderate correlation for ${\mathrm{SO}}_{2}$ is found for both females and males (r=0.5 with $p\text{-}\text{value}=1\times 10^{-12}$ and $3\times 10^{-09}$, respectively).

**Table 7:** Pearson’s correlation coefficient r and corresponding p-value for hemodynamic properties (bold 0.5≤|r|<0.7 moderate correlation, italics 0.3≤|r|<0.5 low correlation, no emphasis 0≤|r|<0.3 no correlation, and bold italics p-value≥0.05.

		${\mathrm{HbO}}_{2}$ (μM)		HHb (μM)		tHb (μM)		${\mathrm{SO}}_{2}$ (%)	
		Female	Male	Female	Male	Female	Male	Female	Male
Age	r	−0.2	0.2	**−0.5**	*−0.3*	*−0.4*	0.0	**0.5**	**0.5**
	p-value	$9\times 10^{-03}$	$5\times 10^{-02}$	$1\times 10^{-12}$	$3\times 10^{-04}$	$3\times 10^{-06}$	$9\times 10^{-01}$	$1\times 10^{-12}$	$3\times 10^{-09}$
BMI z-score	r	*−0.4*	0.0	**−0.6**	*−0.4*	**−0.5**	−0.2	*0.4*	*0.4*
	p-value	$4\times 10^{-06}$	$7\times 10^{-01}$	$4\times 10^{-15}$	$8\times 10^{-06}$	$1\times 10^{-10}$	$4\times 10^{-02}$	$5\times 10^{-08}$	$1\times 10^{-07}$
Head circ.	r	−0.2	0.1	**−0.5**	*−0.3*	*−0.3*	−0.1	*0.4*	**0.5**
	p-value	$9\times 10^{-03}$	$2\times 10^{-01}$	$4\times 10^{-10}$	$3\times 10^{-05}$	$1\times 10^{-05}$	$5\times 10^{-01}$	$4\times 10^{-09}$	$1\times 10^{-09}$

Low inverse correlation with BMI z-score is found for ${\mathrm{HbO}}_{2}$ in females (r=−0.4 with $p\text{-}\text{value}=4\times 10^{-06}$), whereas no correlation is observed in males. A moderate inverse correlation (r=−0.6 with $p\text{-}\text{value}=4\times 10^{-15}$) for HHb is observed in females, and a low inverse correlation (r=−0.4 with $p\text{-}\text{value}=8\times 10^{-06}$) in males. There is a moderate inverse correlation (r=−0.5 with $p\text{-}\text{value}=1\times 10^{-10}$) with BMI z-score for tHb in females but no correlation in males. ${\mathrm{SO}}_{2}$ has a low correlation with BMI z-score for both females and males (r=0.4 for both groups with $p\text{-}\text{value}=5\times 10^{-08}$ and $1\times 10^{-07}$, respectively).

No correlation with head circumference for ${\mathrm{HbO}}_{2}$ is observed for both females and males. Moderate inverse correlation (r=−0.5 with $p\text{-}\text{value}=4\times 10^{-10}$) is found for HHb in females and low inverse correlation (r=−0.3 with $p\text{-}\text{value}=3\times 10^{-05}$) in males. There is a low correlation (r=0.3 with $p\text{-}\text{value}=1\times 10^{-05}$) for tHb in females but no correlation in males. There is a low correlation (r=0.4 with $p\text{-}\text{value}=4\times 10^{-09}$) for ${\mathrm{SO}}_{2}$ in females and moderate correlation (r=0.5 with $p\text{-}\text{value}=1\times 10^{-09}$) in males.

### 3.5 Precision of the Estimates of Optical and Hemodynamic Parameters of the Measured Subjects

**Fig. 6:**
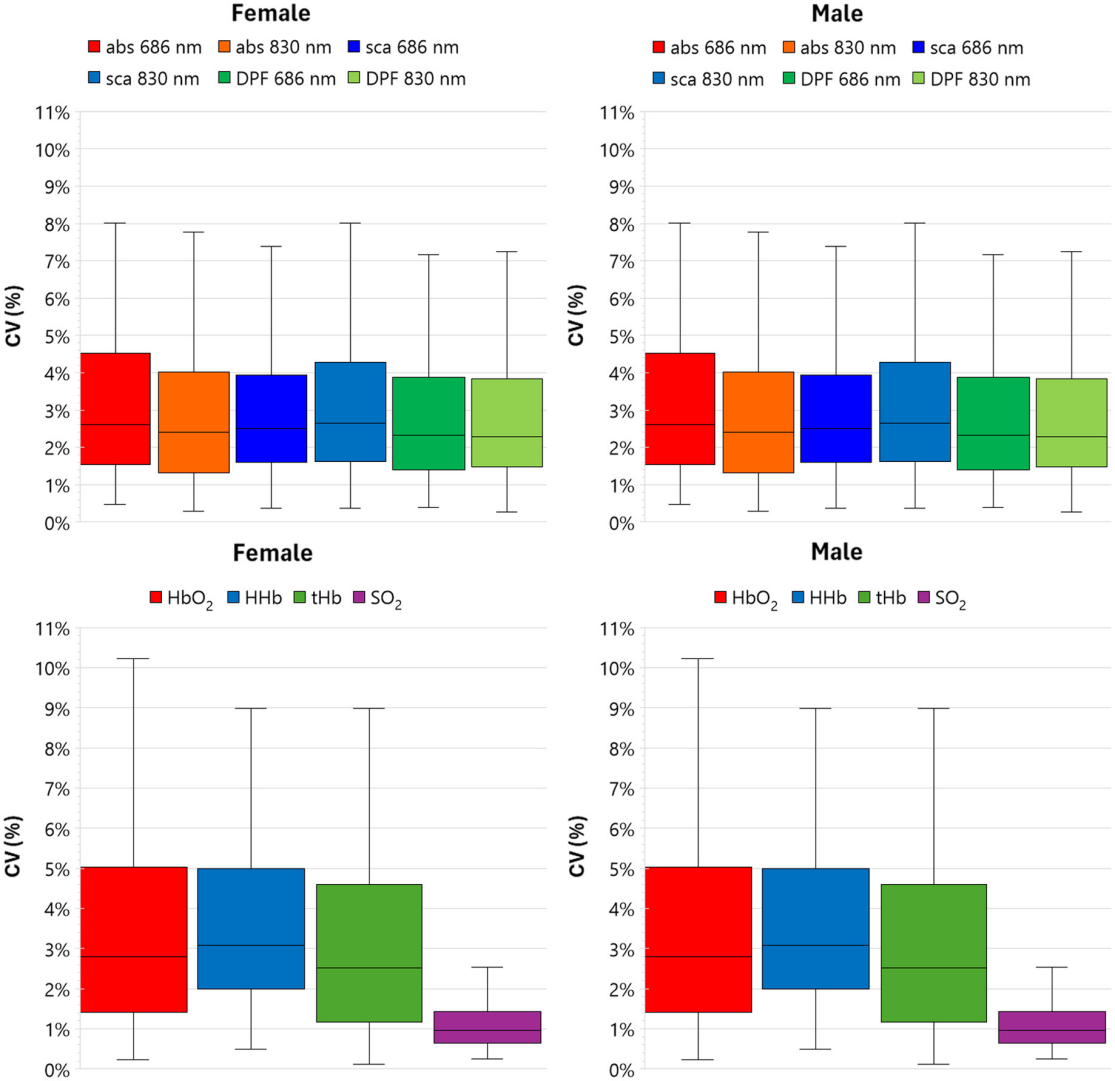
CV over five repositionings for optical parameters (top row) and hemodynamic parameters (bottom row). Left column: female; right column: male.

The precision of measured optical and hemodynamic properties was evaluated by calculating for each subject the coefficient of variation CV(x)=100σ(x)/m(x), where x is the variable under study (e.g., $\mu_{s}^{\prime }$ or ${\mathrm{HbO}}_{2}$), m(x) is the average, and σ(x) is the standard deviation of the five repositionings. Figure 6 shows the boxplots of the CV for optical properties and hemodynamic parameters for all the subjects. The interquartile (25% to 75%) CV for the optical properties is in the range 1% to 5%. The same holds for ${\mathrm{HbO}}_{2}$, HHb, and tHb, whereas noticeably, ${\mathrm{SO}}_{2}$ show a dispersion <2%.

## 4 Discussion

TD NIRS measurements on a large cohort of pediatric patients were performed in a hospital. Absolute values for baseline optical (absorption coefficient, reduced scattering coefficient, and differential pathlength factor at 686 and 830 nm) and hemodynamic parameters (oxygenated and deoxygenated hemoglobin content, total hemoglobin content, and cerebral oxygen saturation) were shown as a function of age and of demographic variables. The TD NIRS device was operated by the clinicians. Each operator attended a short (10 min) training course to learn the basic functions of the device. No adverse effects on the subjects or malfunctioning of the device were recorded during the measurement campaign.

Subject admission to the hospital was related to different causes, such as endocrinological, neurological, osteoarticular, gastroenterological, infectious, or respiratory. According to the inclusion criteria, subjects were measured just before discharge from the hospital, when clinical parameters were stable, and no treatment was ongoing. Therefore, we reasonably hypothesize no effect of the admission cause on the TD NIRS results. Moreover, at the time of measurements, all subjects were calm and cooperative with normal parameters.

Data reproducibility (i.e., dispersion of values during repositioning in each subject) was good being <5% for most of the measured variables and <2% for ${\mathrm{SO}}_{2}$. Precision after probe replacement is indicated as the first problem in cerebral oximetry by NIRS.^48^ In laboratory settings, the NIRSBOX device demonstrated excellent precision (<1%) after probe replacement on tissue phantoms for both $\mu_{a}$ and $\mu_{s}^{\prime }$, whereas slightly higher values (<3% for optical properties and hemodynamic parameters) were found for *in vivo* measurements of the human muscle.^49^ Then, the dispersion of the hemodynamic values during repositioning reported in this study is consistent with laboratory tests and also with other recently developed CW NIRS cerebral oximeters tested on neonates.^20^^,^^50^ Based on our previous experience with TD NIRS, we expect that similar precision values could be obtained with the TD NIRS device also in neonates and adults.

In analyzing the correlation between optical parameters and anthropometric measurements (BMI z-score, age, and head circumference), several key observations emerge. A general trend of higher absorption values in males compared with females was noted. In addition, this difference appears to increase with age. DPF values demonstrated low to moderate correlations with age and head circumference in both sexes but showed no correlation with BMI z-score. This lack of correlation is likely due to the fact that BMI z-score is already adjusted for age and sex, indicating that cerebral DPF values may be linked to growth indicators such as age and head circumference but not to BMI variations.

These anthropometric differences partially extend to cerebral tissue oximetry parameters. A low to moderate correlation was observed between ${\mathrm{SO}}_{2}$ and BMI z-score, age, and head circumference. Interestingly, tHb was correlated with these anthropometric variables only in females, driven primarily by HHb levels. This suggests that auxological factors, particularly in females, may influence cerebral oxygenation metrics. These findings highlight the importance of considering both growth-related variables and BMI when interpreting reference cerebral oximetry values in pediatric populations. Specifically, older and heavier children tend to exhibit slightly higher cerebral ${\mathrm{SO}}_{2}$ values, whereas younger and lighter children, particularly females, show lower brain tHb content.

Overall, the measured optical and hemodynamic properties show values coherent with the literature data.^33^^–^^36^ These data can be helpful for improving the accuracy of other techniques such as DCS and SCOS that rely on values of the optical parameters to derive estimates of tissue perfusion.^51^ Indeed, the accuracy of the measured quantities strongly depends on the physical model used for data analysis. We (such as the majority of the published papers) have used a simple homogeneous model; therefore, values refer to the average properties of the tissue beneath the probe. We have shown in a previous study on adults^27^ that the use of a TD NIRS device with a homogeneous model can provide values of the absorption coefficient closer to the estimates obtained by a two-layer model for the deep layer than to the estimates for the superficial layer. Moreover, penetration depth in TD NIRS is independent from the source–detector distance, whereas it relates to the photon time-of-flight.^52^ From TD NIRS simulations in a two-layer diffusive medium,^53^ we have recently shown that the influence on the penetration depth of the thickness of the superficial layer can be reduced by including photons with late arrival time at the detector, such as it is normally done when fitting TD NIRS data with the photon diffusion model.^54^ For the above observations, we think that the use of the same source–detector distance for all subjects has minimal influence on the results, despite the different anatomical sizes of the head. Therefore, as human cranial vault thickness in the pediatric population is significantly lower than in adults,^55^ we are confident that the values for hemodynamic parameters (being derived from the absorption coefficient) are more representative of the cortical tissue than the extracerebral tissue. Nonetheless, more accurate modeling (e.g., with numerical Monte Carlo simulations based on 3D anatomical mesh) would provide more robust estimates.^32^^,^^56^

The dispersion over the full cohort of the optical properties (∼20% for $\mu_{a}$, 10% for $\mu_{s}^{\prime }$, and 14% for DPF) and of the hemodynamic parameters (∼20% for ${\mathrm{HbO}}_{2}$, HHb, and tHb) is higher than the dispersion in physiological parameters (∼10% for HTC and Hb), being probably affected by the dispersion in BMI (∼30%). This suggests that individual measurements of optical coefficients and hemodynamic contents should be preferred to the average data taken from the literature. Interestingly, ${\mathrm{SO}}_{2}$ shows an overall reduced dispersion of ∼6% and that is supportive being this the most relevant clinical parameter.

In the derivation of the hemodynamic parameters from the absorption coefficient, we have only considered the contribution of hemoglobin, neglecting contributions from other chromophores such as water and lipids. In general, the water content of normal tissues is typically <80% (except for gray matter, placenta, and fetus).^57^ In the extreme case that water contributes 90% to tissue absorption, the errors in the estimate of hemodynamic parameters are 3%, −21%, −13%, and −9% for HHb, ${\mathrm{HbO}}_{2}$, tHb, and ${\mathrm{SO}}_{2}$, respectively. Water absorption is in fact larger at 830 nm than at 686 nm; therefore, it will mainly affect the estimate of ${\mathrm{HbO}}_{2}$. Lipid absorption at short near-infrared wavelengths is low;^47^ therefore, even 90% of lipids contribute minimally to light absorption at 686 and 830 nm. In this case, in fact, the errors in the estimate of hemodynamic parameters are −1%, −5%, −4%, and −1% for HHb, ${\mathrm{HbO}}_{2}$, tHb, and ${\mathrm{SO}}_{2}$, respectively.

## 5 Conclusion

This study provided baseline values for optical and hemodynamic parameters in a large cohort of healthy pediatric subjects with good precision, providing a foundation for future investigations into clinically relevant deviations in these parameters. Although we have observed some correlations of optical and hemodynamic properties with auxological parameters, we do believe that more data are needed to draw robust inferences.

## Data Availability

The codes and datasets generated during and analyzed during the current study are available from the corresponding author upon reasonable request.
